# Supplementary figures and images for: Comparison of two multi-trait association testing methods and sequence-based fine mapping of six additive QTL in Swiss Large White pigs
Source: BMC Genomics. 2023 Apr 10;24:192. doi: 10.1186/s12864-023-09295-4 (PMC10084639; doi:10.1186/s12864-023-09295-4)

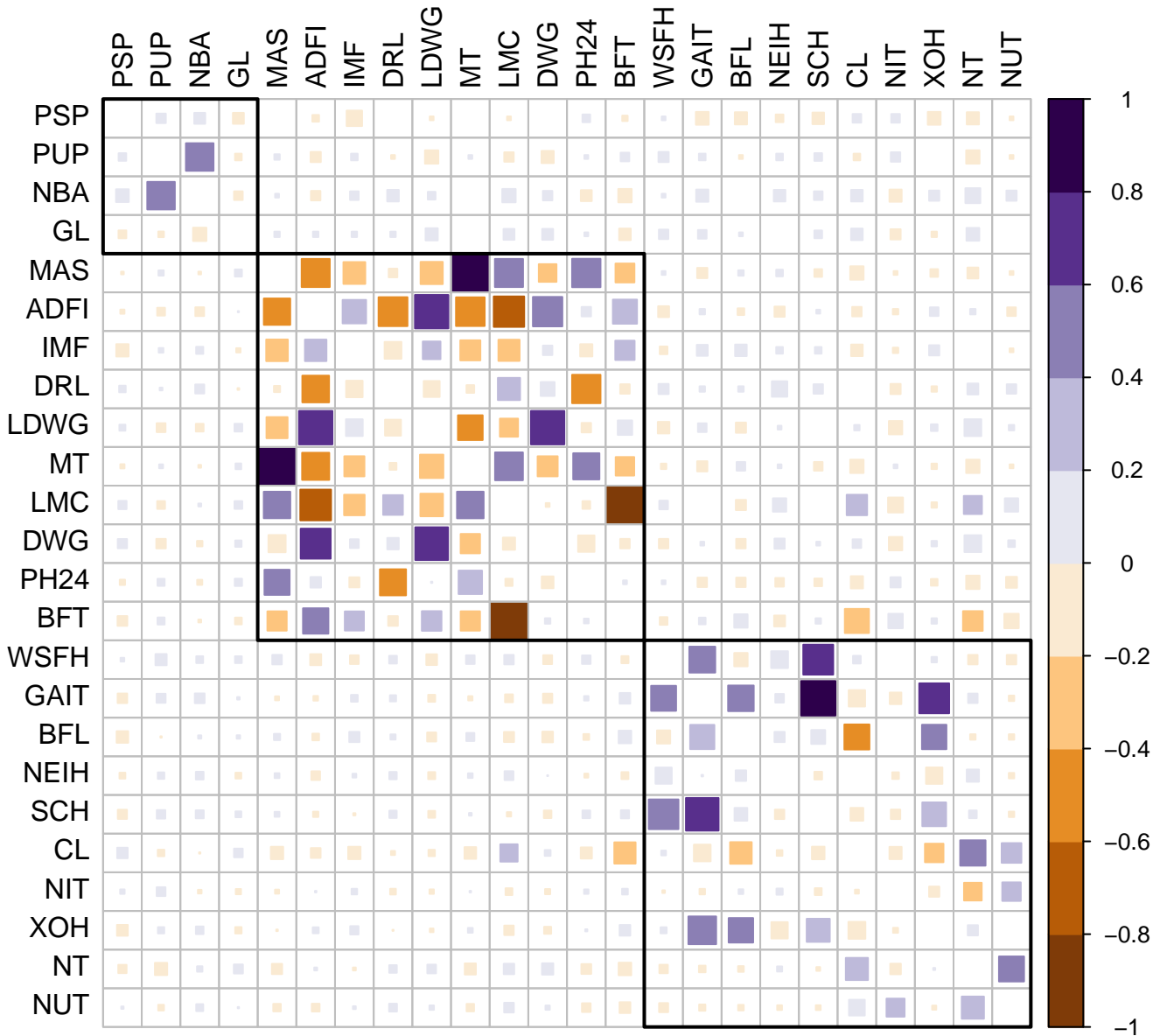

Supplement: Supplementary file 1 — Additional file 1. Correlations between the 24 traits. In the upper and lower triangles are correlations between signed t values, and between deregressed breeding values, respectively. Shades of purple and orange indicate positive and negative correlation coefficients, respectively. The three rectangulars are defining reproduction, production, and conformation groups of traits. [file 12864_2023_9295_MOESM1_ESM.pdf]

Reproduction traits

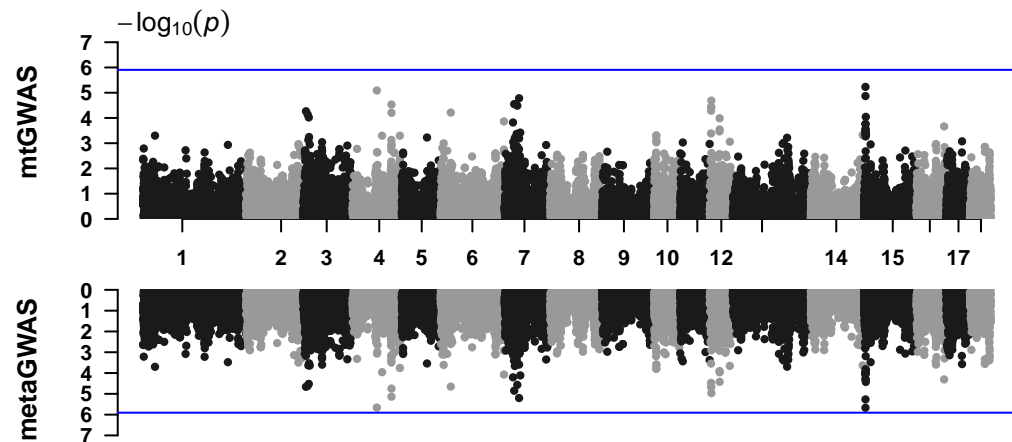

Conformation traits

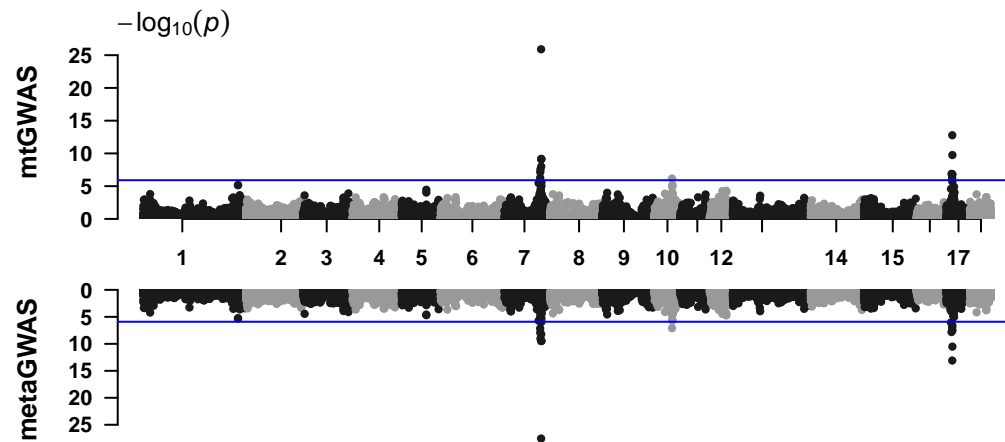

Production traits

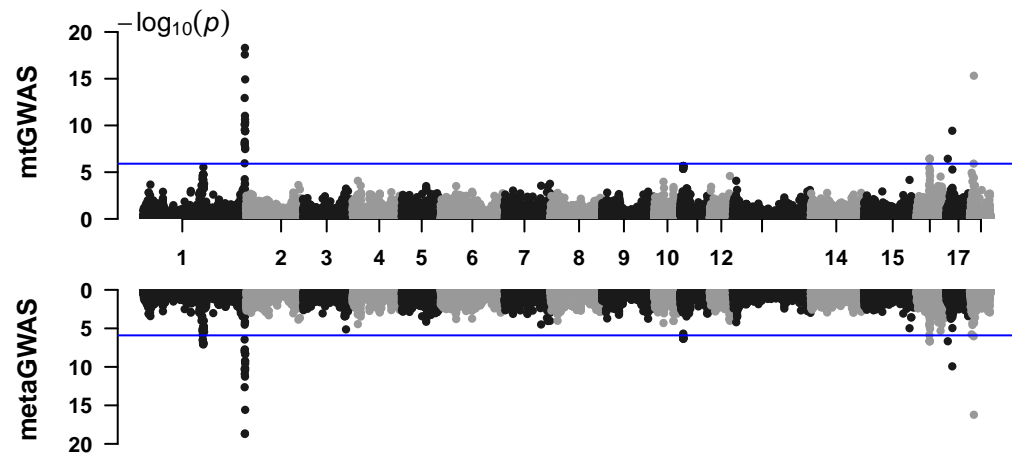

Altogether

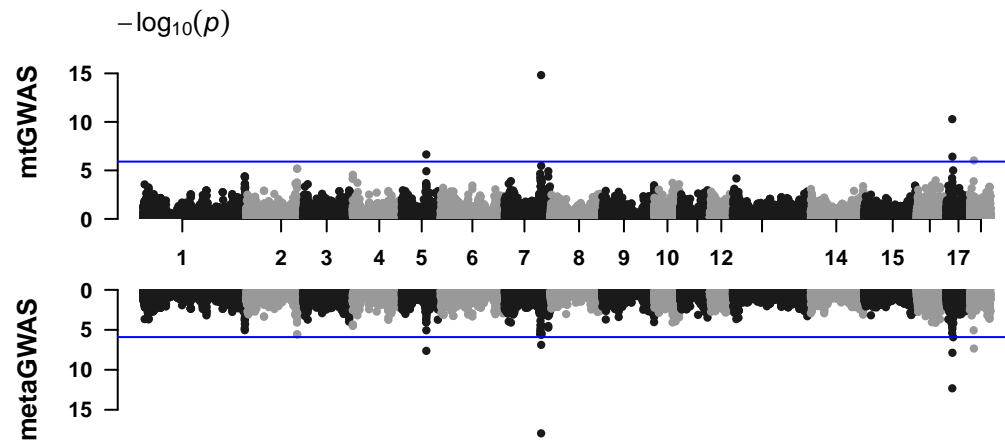

Supplement: Supplementary file 4 — Additional file 4. Manhattan plots of multi-trait GWAS (upper) and meta-analyses GWAS (bottom) based on array genotypes. Suggestive line is at 5.9. [file 12864_2023_9295_MOESM4_ESM.pdf]

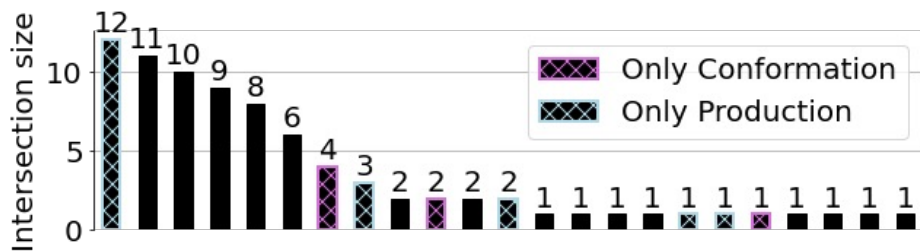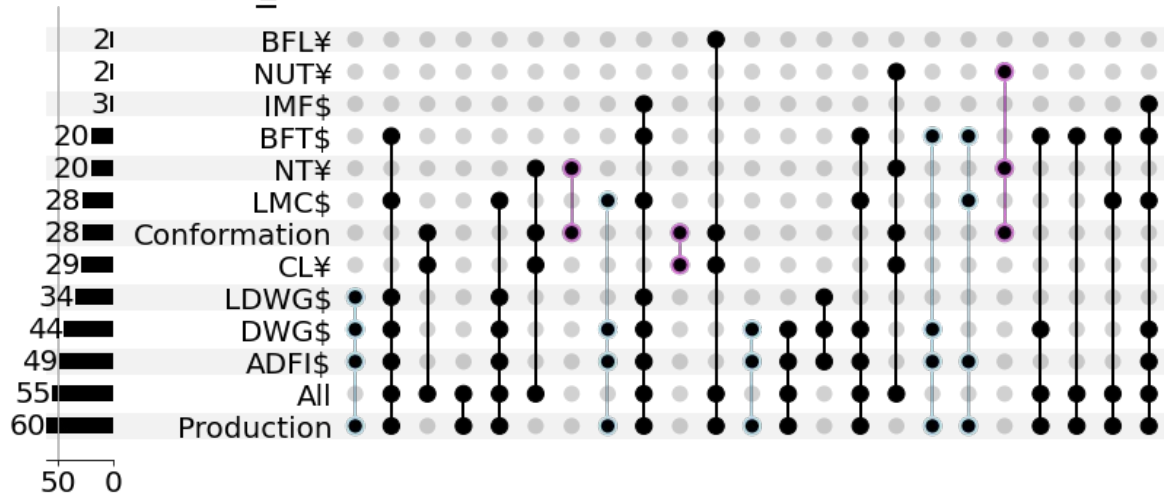

Supplement: Supplementary file 9 — Additional file 9. Number of significantly associated pleiotropic variants in the meta-analyses GWAS within groups using all possible samples and stGWAS of individual traits. The groups are denoted as $ (production group) and ¥ (conformation group). The single traits are: BFL - Bent to pre-bent curve of forelegs; NUT - Number of underdeveloped teats; IMF - Intramuscular fat content in MAS; BFT - Back fat thickness; NT - Number of teats (both sides); LMC - Lean meat content; CL - Carcass length; LDWG - Lifetime daily weight gain; DWG - Daily weight gain on test; ADFI - Average daily feed intake. [file 12864_2023_9295_MOESM9_ESM.pdf]

# PCA of panel and array samples

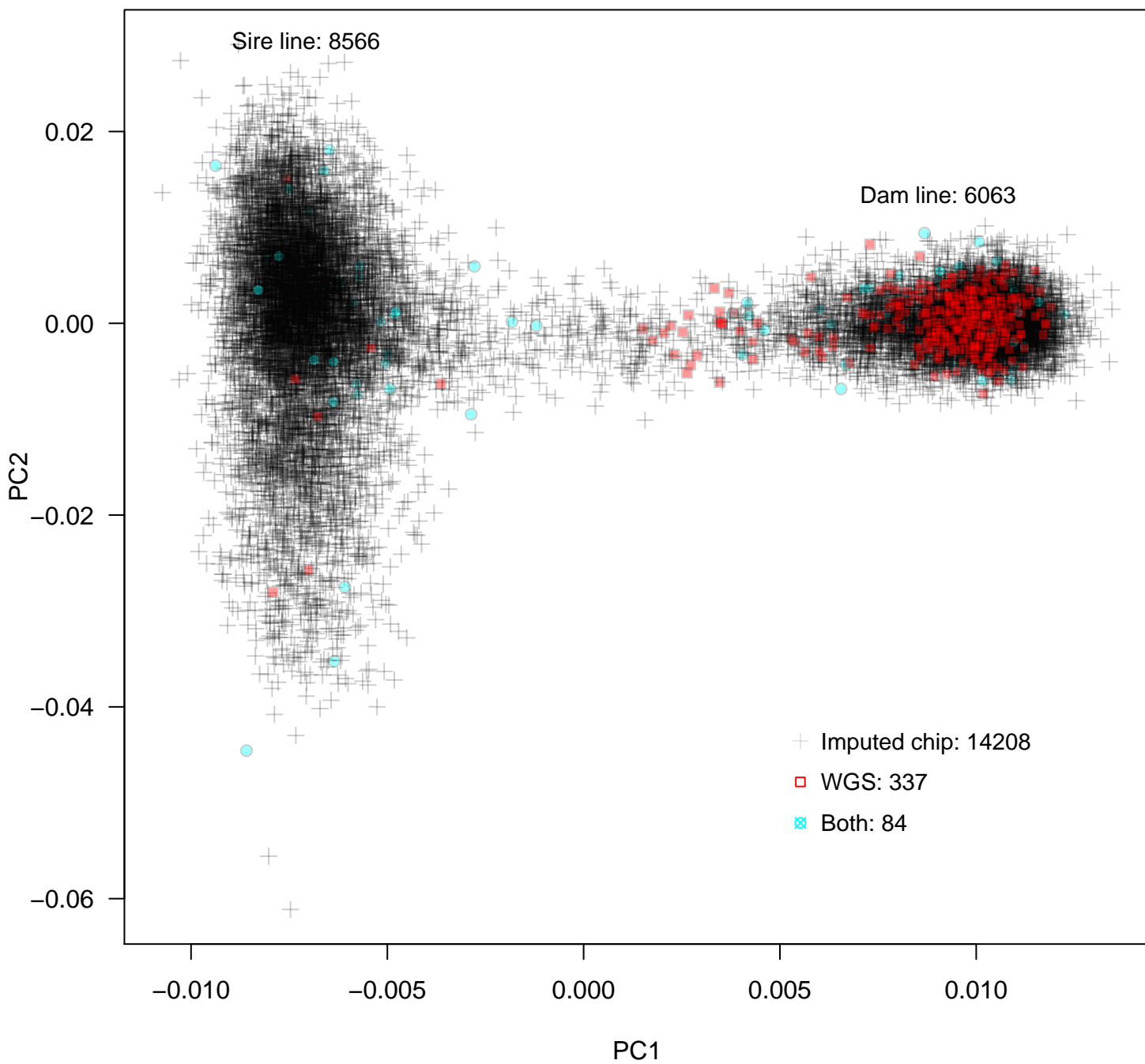

Supplement: Supplementary file 10 — Additional file 10. PCA plot of reference panel samples (red and blue) and the target samples with array-genotypes (black +). First and second principal components captured 7.55 and 0.83% of the genomic variation. [file 12864_2023_9295_MOESM10_ESM.pdf]

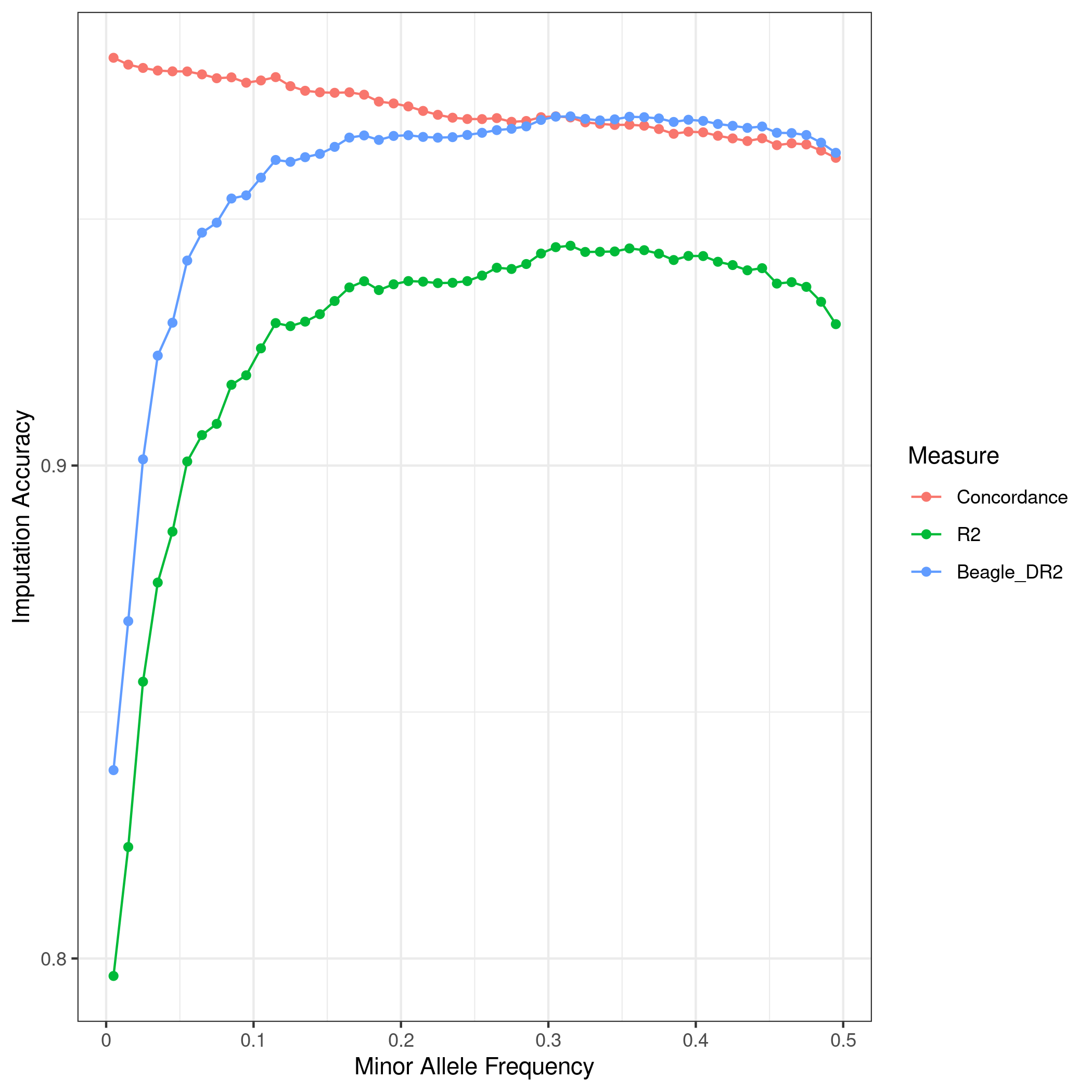

Supplement: Supplementary file 11 — Additional file 11. Accuracy of imputation to whole-genome sequence versus minor allele frequency. Dosage R2 values from Beagle, and empirical measures of concordance and accuracy (R2 ) derived in 5 cross-validation sets, were assessed in 421 animals based on 44,733 downsampled chip genotypes. [file 12864_2023_9295_MOESM11_ESM.png]

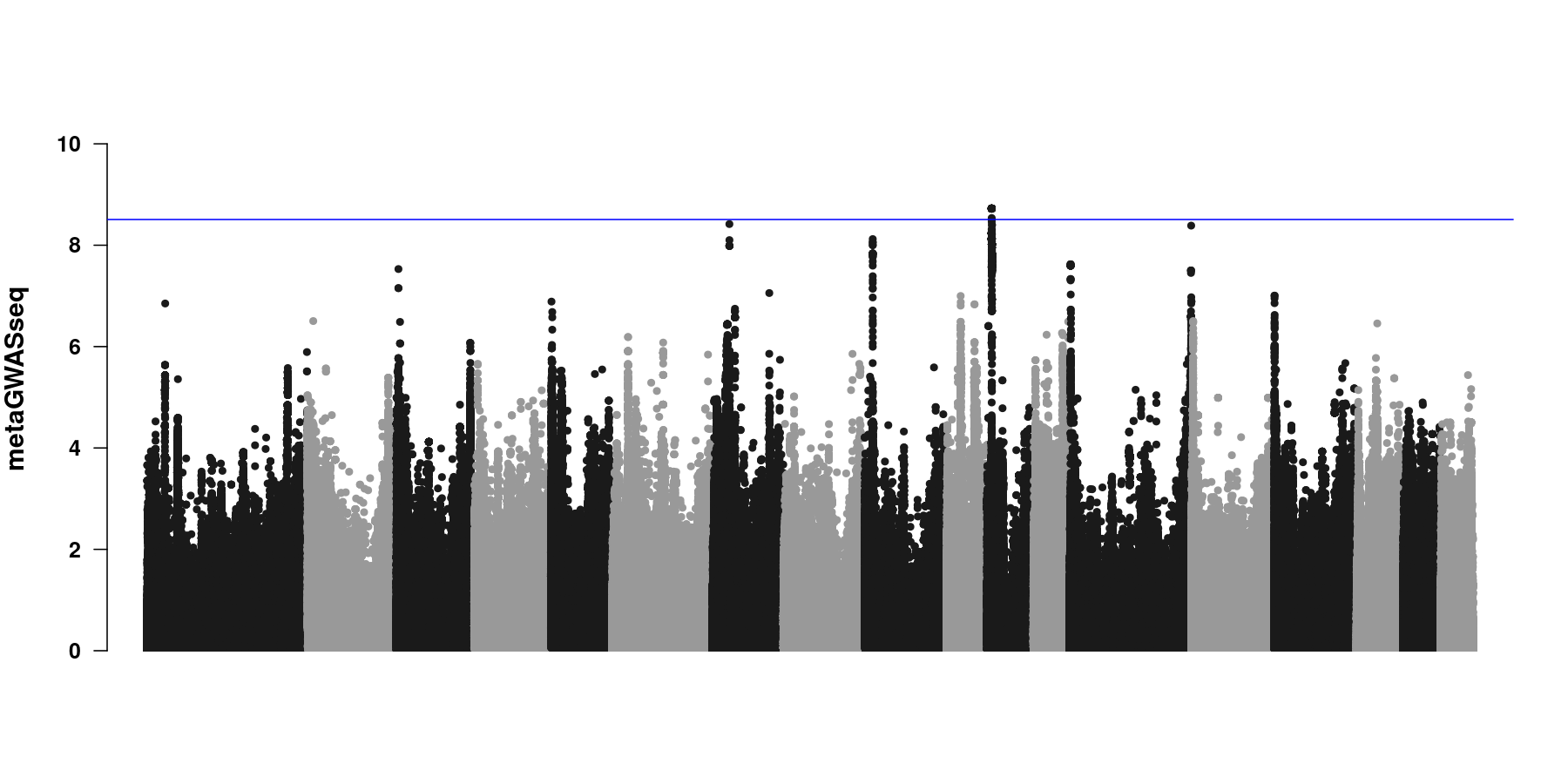

Supplement: Supplementary file 13 — Additional file 13. Manhattan plot of multi-trait meta-analyses GWAS of all 24 traits after fitting the 6 QTL as covariates, which resulted in loss of the peaks. Suggestive line is at 8.5. [file 12864_2023_9295_MOESM13_ESM.tiff]
