# Supplementary material for: Comparison of two multi-trait association testing methods and sequence-based fine mapping of six additive QTL in Swiss Large White pigs
Source: BMC Genomics. 2023 Apr 10;24:192. doi: 10.1186/s12864-023-09295-4 (PMC10084639; doi:10.1186/s12864-023-09295-4)
Supplement: Supplementary file 2 — Additional file 2. Manhattan plots of single-trait GWAS based on array genotypes. Each page contains 4, 10 and 10 plots for traits from reproduction, conformation and production groups, respectively. Blue suggestive line is at 5.9. [file 12864_2023_9295_MOESM2_ESM.pdf]

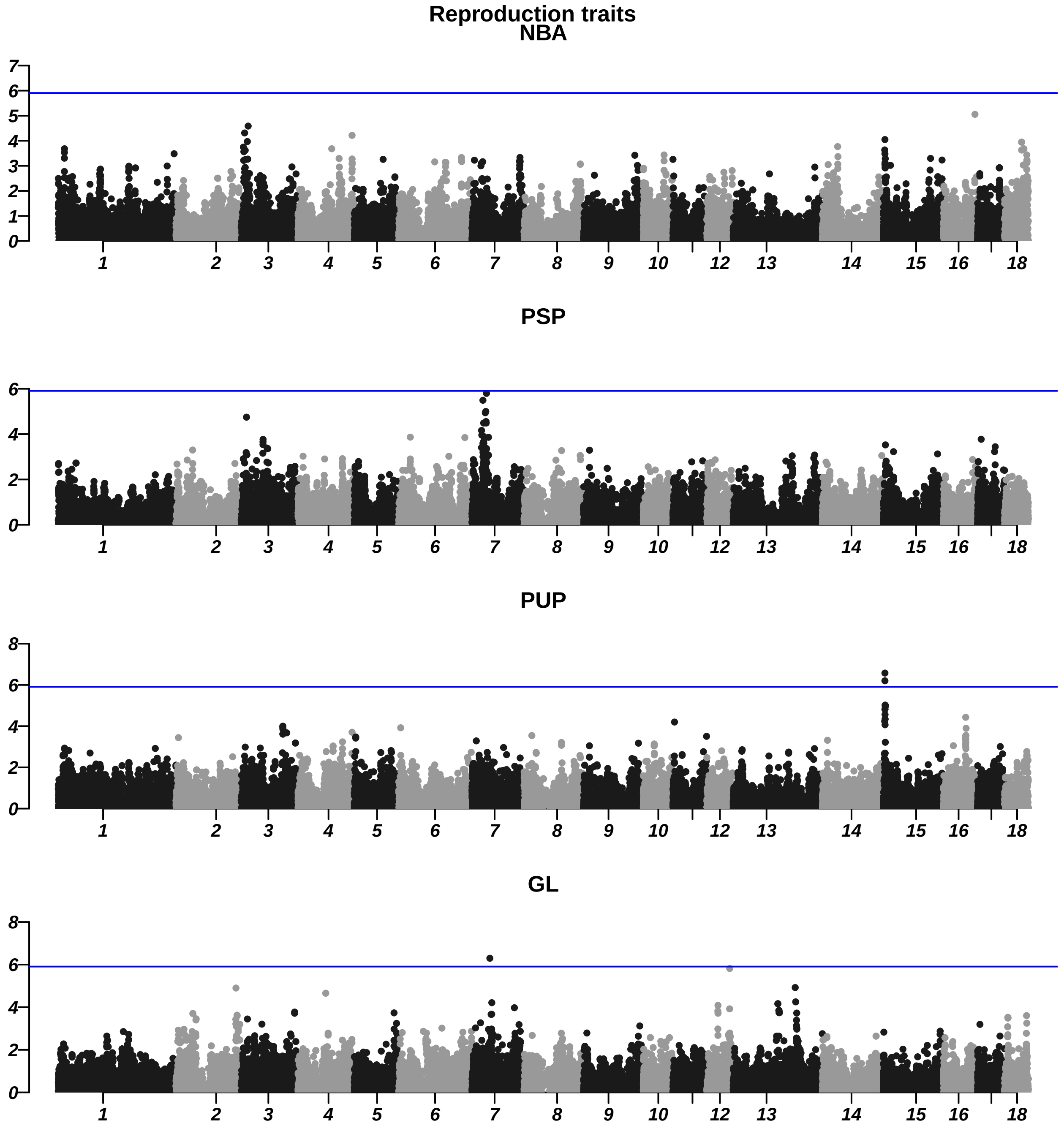
Reproduction traits
NBA
PSP
PUP
GL
1
2
3
4
5
6
7
8
9
10
12
13
14
15
16
18

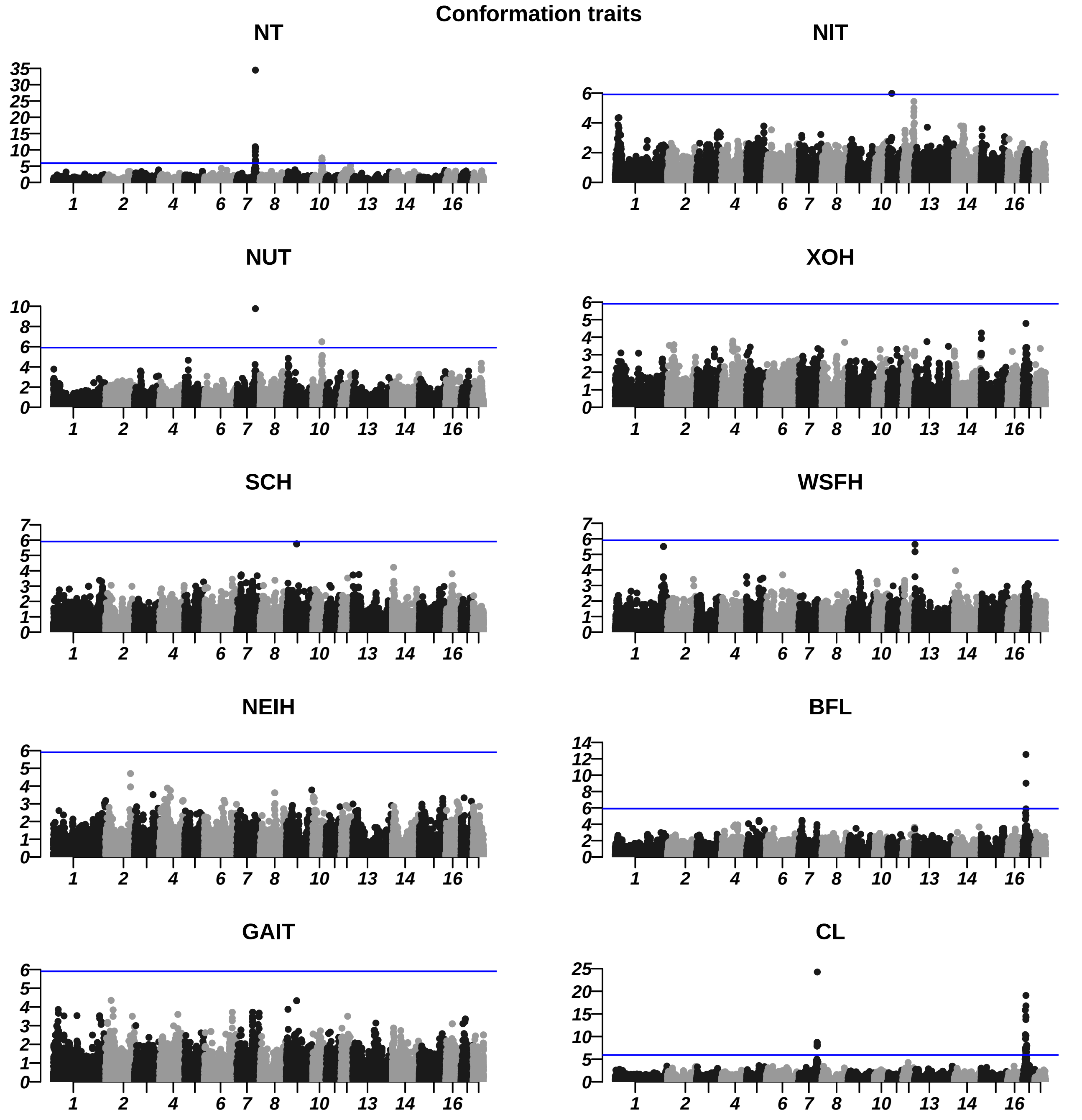
Conformation traits
NT
NIT
NUT
XOH
SCH
WSFH
NEIH
BFL
GAIT
CL
1
2
4
6
7
8
10
13
14
16

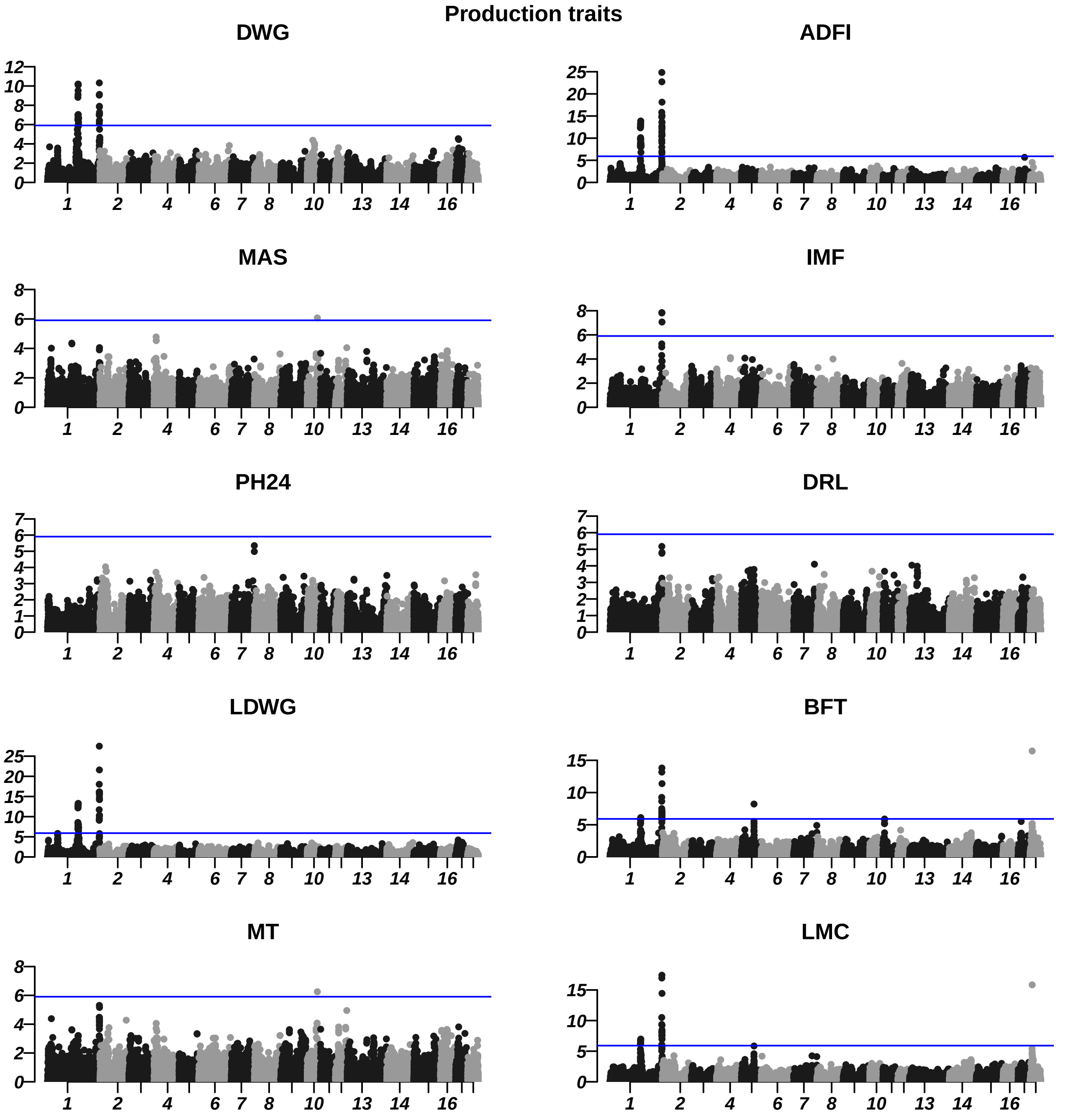
Production traits
DWG
ADFI
MAS
IMF
PH24
DRL
LDWG
BFT
MT
LMC
1
2
4
6
7
8
10
13
14
16
